# Vegetation dynamics inside Mediterranean vineyards: A dataset for tracking changes using unmanned aerial vehicles

**DOI:** 10.1016/j.dib.2026.112660

**Published:** 2026-03-09

**Authors:** Martin Faucher, Guilhem Brunel, Anice Cheraiet, Clément Enard, Denis Feurer, Fabrice Vinatier, Léo Garcia

**Affiliations:** a ABSys, Univ. Montpellier, CIRAD, INRAE, Institut Agro, Montpellier, France; b ITAP, Univ. Montpellier, INRAE, Institut Agro, Montpellier, France; c LISAH, Univ. Montpellier, AgroParisTech, INRAE, Institut Agro, IRD, Montpellier, France

**Keywords:** Teledetection, Agroecology, Cover crops, Viticulture, Multispectral imaging, Structure from motion (SfM)

## Abstract

Service crops are grown to provide ecosystem services in viticulture, but their adoption remains limited due to their competition with grapevine for soil resources. To identify trade-offs between services, the effect of service crops management strategies on grapevine performances still need further research. This dataset presents data from two experiments conducted to study the effect of service crops management on soil resources and grapevine performances. The inter-row vegetation was sampled in two Mediterranean vineyards using quadrats for biomass estimation. In addition, an unmanned aerial vehicle (UAV) was regularly flown over the vineyards for a period spanning more than four years in total over the two vineyards. The dataset presented here includes both raw data acquired during fieldwork and processed data derived from this raw inputs. The raw data consists of image series captured by two UAVs during each flight campaign, including RGB and multispectral imagery. Images were acquired between 2021–06–10 and 2022–07–29 for the first vineyard, and between 2023–06–08 and 2025–03–12 for the second vineyard. Based on these raw data, the processed data comprises spatial vectors, raster layers, and dense point clouds generated from UAV images using a Structure from Motion (SfM) photogrammetry workflow, at a 5 cm spatial resolution. The raster layers and dense point clouds provide specific information on vineyard characteristics for each UAV flight date, including elevation, vegetation indices, visible and near-infrared reflectance, and canopy height. In addition, the processed data include measurements of vegetation dry biomass, as well as separate measurements of dry biomass and leaf area measured for selected service crops species. This dataset can be reused for the calibration and/or evaluation of classification algorithms aimed at discriminating vines from the inter-row vegetation, or as part of a larger dataset to explore relationships between remotely-sensed vegetation indices and field-measured vegetation biomass or surface.

Specifications Table

**Table:** 

Subject	Earth & Environmental Sciences
Specific subject area	This dataset is focused on inter-row vegetation evolution inMediterranean vineyards, tracked by UAV surveys performed on different dates over the same vineyards.
Type of data	Image (.png, .tif), raw, stored in folders compressed in .zip Spatial Rasters (.tif), processed Spatial Vectors (.gpkg), processed Dense point clouds (.ply), processed
Data collection	Images were collected using a DJI Phantom Pro drone equipped with both a RGB and a multispectral camera. Agisoft Metashape Professionnal was used to align the images and produce the rasters and point clouds included in the dataset. Vegetation samples were collected using 50×50 cm quadrats and subsequently placed into a drying oven at 60 °C for 48 h then weighed using a Precisa LX2200C balance. Individuals of several plant species were additionally collected, and their leaves scanned using the WinFOLIA software, then they were placed in the drying oven. At the end of the drying period, leaves and stems were separately weighted using a Mettler Toledo ML2023 balance with a 0.01 g error threshold.
Data source location	Data were collected in vineyards located in southern France near Montpellier (43°32.5243′N, 3°50.8240′E). Data are stored on Research Data Gouv, a remote storage solution curated by the French Department of Research.
Data accessibility	Repository name: Research Data Gouv Data identification number: doi: 10.57745/MXM55R Direct URL to data: https://doi.org/10.57745/MXM55R
Related research article	None

## 1 Value of the Data

• The fine scale imaging of vineyards (i.e., 5 cm resolution) allows for classification of the vegetation in the vineyard inter-rows, and subsequent exploration of its respective dynamics.
• These data are intended primarily for reuse by other researchers, for example in meta-analyses that compare inter-row vegetation dynamics in vineyards under contrasting pedoclimatic conditions, using UAV surveys. The data could also be used to simulate repeated field surveys of vegetation, with the aim of constructing a calibration and evaluation dataset for a vegetation growth model.
• On both vineyards, contrasted agricultural operations were conducted, thus offering an opportunity to compare their effects with limited environmental interferences.
• Both campaigns span an extensive period of time, and offer a reduced timestep during key vine development stages.
• In light of climate change, providing data from agroecological vineyards promotes the development of knowledge on sustainable agricultural practices, alleviating the environmental pressures on viticulture and reducing its environmental impact.

## 2 **Background**

In the context of climate change and increasing socioeconomic pressures, vineyard management practices of the northern Mediterranean basin are facing growing demands to transition toward more sustainable practices [1]. However, under the dry Mediterranean climate, associating herbaceous vegetation with grapevine may lead to competition for water between vines and the adjacent non-cultivated vegetation [2]. Assessing the effect of such practices on vegetation levels within vineyards can be hard to obtain at an appropriate spatial and temporal scale due to the numerous variables involved [3]. By using UAV flight campaigns to monitor vegetation dynamics over time, this study aims to build on previous work that successfully tracked vine development using this type of data [[4], [5], [6]], and to extend this framework to the inter-row vegetation, in addition to the well-established monitoring of vine growth.

## 3 Data Description

**Fig. 1:**
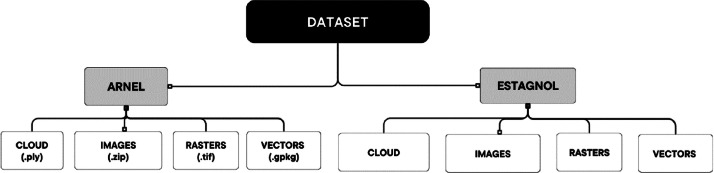
General organization of the dataset, and associated file formats. The “Images” subfolders contain compressed folders of .png and .tif images for each date and each camera (i.e., RGB and multispectral) separately. Each folder named with a white background can be downloaded as a whole from the data repository.

The dataset is composed of two main folders referring to the two vineyards where the data were collected, subsequently named ESTAGNOL and ARNEL. Both folders follow the same structure constituted of subfolders defined by the type of data they contain: Cloud, Image, Rasters, and Vectors (Fig. 1). Each subfolder is stored as compressed. All files except those referring to time-invariant characteristics are named based on the same template: “Date (YYYY_MM_DD)_Plotname_description.format”. An exception is made for files located in the “Images” subfolders, for which the original file names were kept, but stored in folders named following the template described above. For example, “2022_01_19_Estagnol_DEM.tif” is the raster containing the Digital Elevation Model (DEM) of the Estagnol plot on January 19th of 2022.

Each “Rasters” subfolder is further divided into five subfolders corresponding to the image types, and named using acronyms : Digital Elevation Models (DEM), Digital Surface Models (DSM), Canopy Height Models (CHM), orthomosaics (ORT), and rasters of vegetation indices (VEG).

## 4 Experimental Design, Materialand Methods

### 4.1 Location

**Fig. 2:**
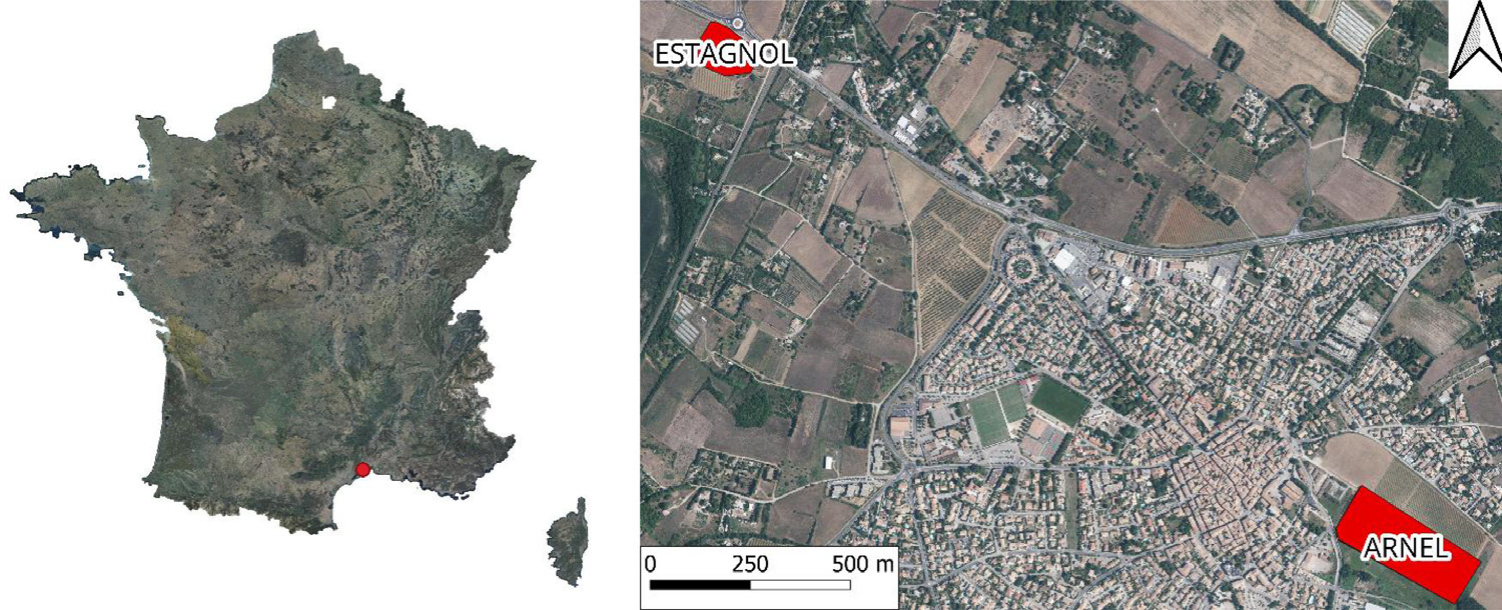
The general location of the vineyards (shown in red) is shown relative to mainland France, and to each other within the town of Villeneuve-lès-Maguelonne.

Data were collected in two vineyards located in southern France in Villeneuve-lès-Maguelone, hereafter named the Estagnol vineyard (43°32.5243′N, 3°50.8240′E), and the Arnel vineyard (Fig. 2).

### 4.2 UAV flights

UAV flights were performed using a DJI Phantom 4 Pro v2 and a DJI Phantom 4 M drones for RGB and multispectral images, respectively. All images were acquired using drones built-in cameras. The DJI Phantom 4 M drone captures Red Edge (730 ± 16 nm) and Near Infrared (840 ± 26 nm) wavelengths along with the Red (650 nm ± 16 nm), Green (560 nm ± 16 nm), and Blue (450 nm ± 16 nm) wavelengths also captured by the DJI Phantom 4 Pro v2 drone. The drones were flown 30 and 50 m over the vineyards at a nominal speed of 2.8 m/s, for the Estagnol and Arnel vineyards respectively. The cameras were set to capture images at a 70° and 90° tilt for RGB and multispectral imagery, respectively, with an 80 % frontal and 70 % side overlap. Flight missions were planned using the manufacturer apps (DJI Go 4 and DJI Ground Station Pro, Nanshan District, Shenzhen, China).

On the Estagnol vineyard, UAV campaigns were conducted on clear-sky days from 2021–06–10 to 2022–07–29. On the Arnel vineyard, UAV campaigns were conducted on clear-sky days from 2023–06–08 to 2025–03–12.

7 and 12 Ground Control Points (GCPs) were placed on the Estagnol and Arnel vineyards respectively, their precise GNSS location were retrieved, and stored inside a dataframe as part of the post-alignment procedure performed during the processing of the images collected during the UAVs campaigns (Table 1). GCPs were placed on the Estagnol vineyard on three separate campaigns, 2021–12–07, 2022–01–19, and 2022–03–16, and on the Arnel vineyard on the 2025–03–12 campaign.

**Table 1:** Summary of the information related to the two vineyards surveyed by the unmanned aerial vehicles (UAVs).

Vineyard plot	Estagnol	Arnel
Surface surveyed (ha)	1.5	4.8
Period of time covered	2021–06–10 to 2022–07–29	2023–06–08 to 2025–03–12
Number of flights performed	17	14
Number of Ground Control Points (GCPs)	7	12

### 4.3 UAV-derived data

**Fig. 3:**
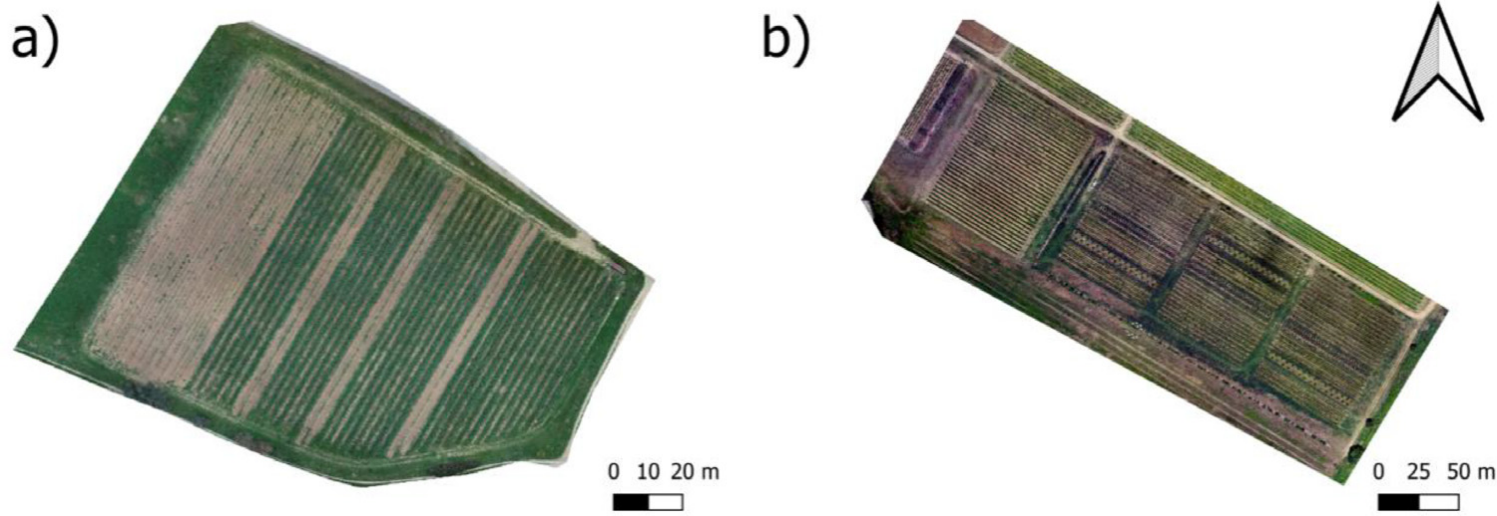
Examples of orthomosaics produced for the a) Estagnol and b) Arnel vineyard.

DEM, orthomosaics and dense point clouds were processed using Agisoft Metashape Professional (Agisoft LLC, St Petersburg), following the Time-SIFT method developed by Feurer et al. (2018) [7]. UAV flight campaigns were loaded into the software and merged into a single chunk. Then photos were aligned with 40,000 key points and 4000 tie points. GCPs were then added to the point cloud resulting from the alignment procedure. Finally, the information for each date was processed separately to produce the dense point cloud, the DEM, and the complete orthomosaic raster of the vineyard (Fig. 3). For each date, multispectral images were only used to produce an orthomosaic raster. No radiometric calibration or correction was applied to multispectral images. Raster resolution was set to 5 cm as a trade-off between accuracy and file size. All exported files were georeferenced using the WGS 84 / UTM zone 31 N (EPSG:32,631) projection.

A script automatizing most of the operations described by the Time-SIFT method was applied to our data. The script used is available publicly and can be found online [8]. Only the initial data loading and the correction of positions using the ground control points were completed manually. Correction of positions using the GCPs consisted in verifying whether the position of those points on the point cloud coincided with the corresponding visual markers set up on the field.

**Fig. 4:**
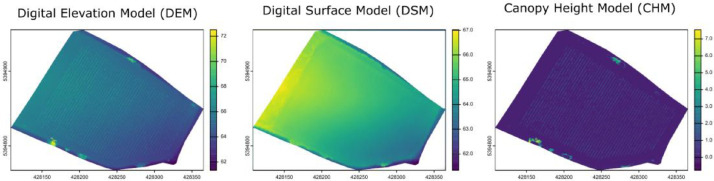
Example of a raster of Digital Elevation Model (DEM), used for separating Digital Surface Model (DSM) from Canopy Height Model (CHM). The rasters presented here correspond to the Estagnol vineyard.

Dense point clouds were used to produce DSMs using the CloudCompare Software v2.13.2. First, the Cloth Simulation Filtering plugin [9] was used to separate ground and off-ground points. The scenes parameter was set to ‘Flat’, cloth resolution was set to 0.1 and class threshold to 0.05. Then, the point cloud containing the ground points was rasterized with the Z coordinate as cell value, and empty cells were filled by kriging. Finally, the resulting DSMs were stored in .tif files, and used as ground references to calculate CHM, set for each date as the difference between the DEM and the DSM (Fig. 4).

**Fig. 5:**
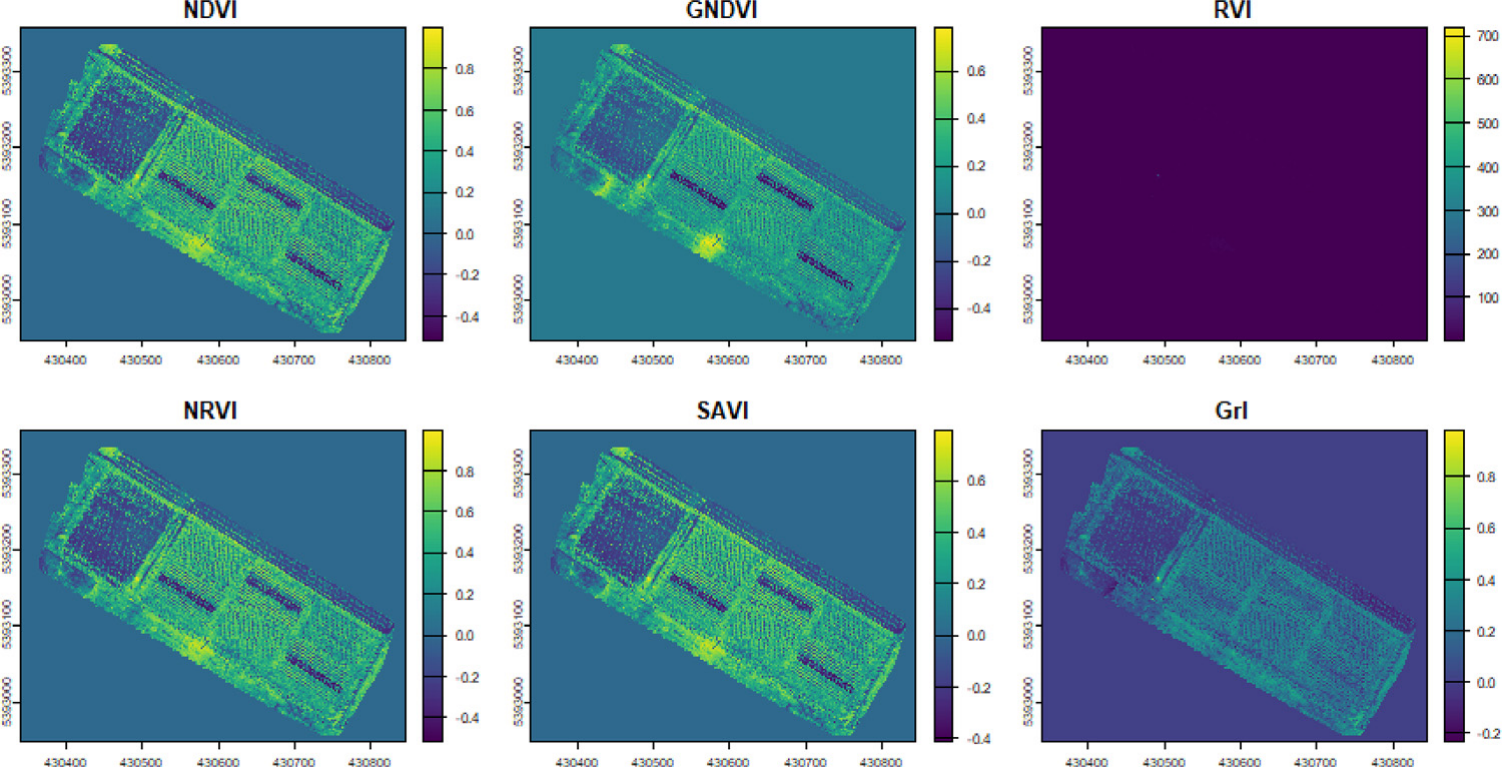
Example of a multiband raster of vegetation indices computed for the Arnel vineyard using the orthomosaic resulting from using the Time-SIFT method on multispectral images collected the same day.

The vegetation indices stored in the multiband VEG rasters were computed using the multispectral orthomosaic rasters produced by opening the rasters with R software and the ‘terra’ package (Table 2; Fig. 5).

**Table 2:** Vegetation indices stored in the multiband rasters inside the VEG folders and associated formulas. NIR, R, and G correspond to the Near Infrared (840 ± 26 nm), Red (650 nm ± 16 nm), and Green (560 nm ± 16 nm) wavelengths, respectively. L corresponds to the soil adjustment factor and was set to −0.2.

Vegetation index (VI)	Formula
Normalized Difference Vegetation Index (NDVI)	$\frac{NIR-R}{NIR+R}$
Green Normalized Difference Vegetation Index (GNDVI)	$\frac{NIR-G}{NIR+G}$
Ratio Vegetation Index (RVI)	$\frac{NIR}{R}$
Normalized Ratio Vegetation Index (NRVI)	$\frac{RVI-1}{NIR+1}$
Soil-Adjusted Vegetation Index (SAVI)	$\frac{NIR-R}{NIR+R+L}\left(1+L\right)$
Greenness Index (GrI)	$\frac{G-R}{G+R}$

### 4.4 Field data

On the Estagnol field, vegetation samples were collected on the 2022–01–19, 2022–02–16, 2022–03–16, 2022–04–06, and 2022–05–18. On the Arnel field, vegetation samples were collected on 2023–05–31, 2024–03–18, 2024–05–13, and 2025–03–12.

For each dataset, vegetation was sampled in 50×50 cm quadrats, where vegetation was removed and collected into paper bags. Sown and spontaneous species were then sorted, and placed in separate paper bags. Vegetation was oven dried at 60 °C for 48 h, then weighed inside the paper bags using a Precisa XB2200C balance. 10 empty paper bags were weighed and the mass of a single paper bag was deducted.

The differentiation of select species during sampling was combined with additional work conducted on all sampling dates for the Estagnol field, and on 2025–03–12 for the Arnel field. For each selected species, 10 additional plants were collected and individually stored into paper bags. Individuals collected were selected in order to have different sizes of the same species. Each plant was separated between leaf and stem parts in the lab. Leaves were scanned using the WinFOLIA software (Regent Instruments, Canada). Leaves and stems were oven dried at 60 °C for 48 h, then separately weighed outside of their paper bags using a Mettler Toledo ML203 balance.

### 4.5 Spatial vectors

**Fig. 6:**
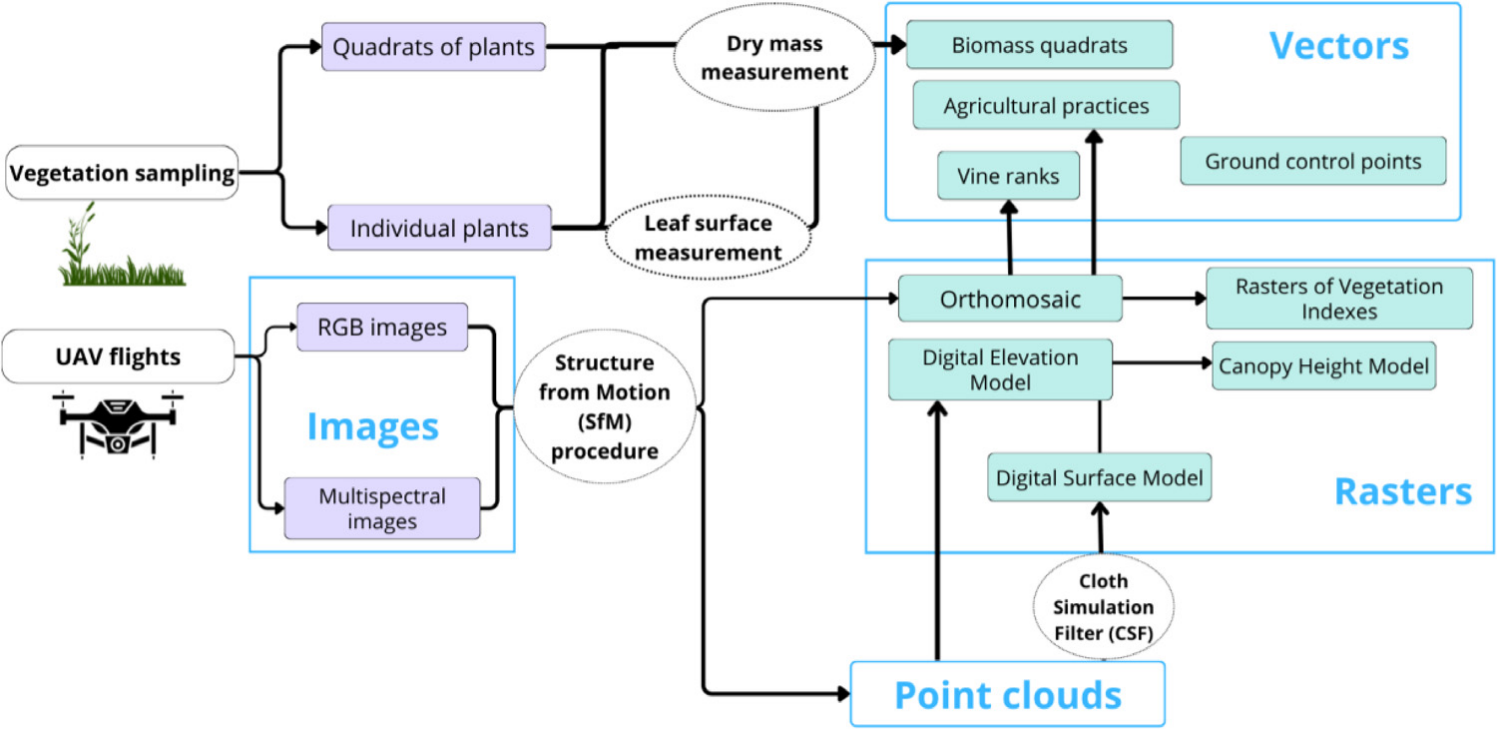
Description of the data collected (in purple) and the data processed (in green). White circles are used to represent the main processing steps, blue squares represent the data types included in the dataset.

For each field, spatial vectors were then created to link field data with geographical coordinates, thus allowing for their comparison with data stored in the point clouds and rasters. Four distinct vectors are provided for each field: “[…]_biomassQuadrats.gpkg” files represent biomass quadrats as polygons, “[…]_agriculturalPractices.gpkg” files represent the agricultural practices as polygons encompassing the areas they were applied, “[…]_ranks.gpkg” files contain the vine ranks stored as polylines, and “[…]_GCP.gpkg” files contain the ground control points used during the processing of the UAV-derived data. Vegetation quadrats vectors were created by generating a 50×50 cm square buffer around the GNSS coordinates taken for each biomass collection, vine rows and agricultural practices were manually drawn based on the orthomosaics produced, and ground control points vectors were automatically created by importing their GNSS coordinates (Fig. 6).

## **Limitations**

For the Estagnol dataset, three UAV campaigns could not be performed on the entire study area due to lack of time. Thus, data based on the 2022–02–16, 2022–02–25, and 2022–05–13 flight surveys do not include part of the vineyard, which might lead to missing data during diachronic studies of areas located in the missing part of the vineyard. Additionally no radiometric correction was applied to multispectral data, which may hinder their use for fine-tuned measurements.

## Ethics Statement

The authors have read and follow the ethical requirements for publication in Data in Brief and confirm that the current work does not involve human subjects, animal experiments, or any data collected from social media platforms.

## CRediT Author Statement

**Martin Faucher:** Conceptualization, Data curation, Formal analysis, Investigation, Software, Writing – Original draft, Visualization; **Guilhem Brunel:** Writing – Reviewing; **Anice Cheraiet:** Writing – Reviewing; **Clément Enard:** Data acquisition; **Fabrice Vinatier:** Software, Writing – Reviewing; **Denis Feurer:** Software, Writing – Reviewing; **Léo Garcia:** Supervision, Conceptualization, Investigation, Methodology, Data curation, Project administration, Funding acquisition.

## Data Availability

DataverseEvolution of vegetation levels in Mediterranean vineyards : comparing field observation data and drone imaging (Original data).

## Appendix Supplementary materials

Image, application 1
